# Evaluation of *Lacticaseibacillus casei* 39 Paraprobiotic in Modulating Inflammatory and Oxidative Pathways in Acetic Acid Induced Ulcerative Colitis

**DOI:** 10.1002/fsn3.70476

**Published:** 2025-07-18

**Authors:** Adem Yavaş, Ecem Akan

**Affiliations:** ^1^ Food Processing Department, Food Quality Control and Analysis Programme, Çine Vocational School Aydin Adnan Menderes University Aydin Türkiye; ^2^ Department of Dairy Technology, Faculty of Agriculture Aydin Adnan Menderes University Aydin Türkiye

**Keywords:** inflammation, paraprobiotic, PI3K/AKT signaling, ulcerative colitis, VEGF/EGF

## Abstract

Ulcerative colitis (UC) is a chronic inflammatory bowel disease marked by mucosal inflammation, oxidative stress, and increased apoptosis. This study investigated the therapeutic effects of UV‐C‐inactivated *Lacticaseibacillus casei* 39 (paraprobiotic) in an acetic acid‐induced UC rat model. The study evaluated its effects on clinical symptoms, inflammatory cytokines, oxidative stress, growth factor expression, PI3K/AKT pathway activation, and apoptotic markers. Paraprobiotic was obtained by UV‐C inactivation of 10^8^ CFU/mL *L. casei* 39. Female Wistar rats were divided into four groups: Control, UC, UC + Probiotic, and UC + Paraprobiotic (*n* = 10/group for clinical, ELISA, and oxidative stress analyses; *n* = 3 pooled/group for Western blot). UC was induced by rectal acetic acid administration, followed by daily oral treatments for 6 days. Clinical signs (DAI, weight change), plasma/tissue cytokines (ELISA, Western blot), oxidative stress (colon TOC/TAC), VEGF/EGF mRNA (qRT‐PCR), and apoptosis/PI3K/AKT pathway proteins (Western blot) were assessed. The UC group showed severe colitis, increased TNF‐α and IL‐6, decreased IL‐10, elevated TOC, reduced TAC, altered VEGF/EGF mRNA, PI3K/AKT activation, and increased apoptosis (Bax/Bcl‐2 ratio, Cytochrome c, Caspase‐9, Caspase‐3). Paraprobiotic treatment improved clinical symptoms, reduced pro‐inflammatory cytokines, and increased anti‐inflammatory cytokines, modulated growth factor expression, reduced oxidative stress, suppressed PI3K/AKT signaling, and downregulated apoptotic markers. TOC decreased 2.10‐fold (*p* < 0.01), TAC increased 5.43‐fold (*p* < 0.001), and Bax/Bcl‐2 ratio and Caspase‐3 expression were significantly reduced (*p* < 0.001 vs. UC; Caspase‐3 also *p* < 0.05 vs. UC + Probiotic). *L. casei* 39 paraprobiotic exhibits potent anti‐inflammatory, antioxidant, and anti‐apoptotic effects in UC, suggesting its promise as a novel, multi‐target therapeutic agent.

## Graphical abstract

UV‐C inactivated *Lacticaseibacillus casei* 39 paraprobiotic suppressed inflammation (↓TNF‐α, IL‐6; ↑IL‐10), oxidative stress (↓TOC, ↑TAC) and apoptosis (↓Bax/Bcl‐2, ↓Caspase‐3/9, ↓cyt c) in an acetic acid‐induced colitis model. It promoted healing by regulating VEGF/EGF expression. It showed a multi‐targeted protective effect by inhibiting PI3K/AKT pathway activation. These findings suggest that *L. casei* 39 paraprobiotic is a promising anti‐inflammatory, antioxidant, and anti‐apoptotic agent in the treatment of ulcerative colitis.

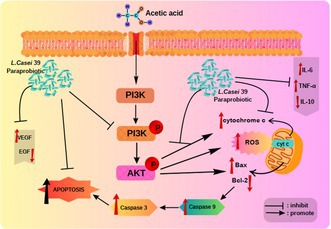

## 1 Introduction

Ulcerative colitis (UC) is a major form of inflammatory bowel disease (IBD) and poses a significant global health challenge due to its chronic and relapsing nature. Characterized by continuous inflammation and ulceration of the colonic mucosa, UC severely diminishes patients' quality of life and increases the risk of colorectal cancer (Liashev et al. 2024; Segal et al. 2021; Yan et al. 2023). The complex etiology of UC is understood to involve a dysregulated interplay between genetic predispositions, environmental factors, an aberrant immune response, and alterations within the gut microbiota composition and function (He et al. 2022; Quansah et al. 2023). This interplay results in a compromised intestinal epithelial barrier and sustained inflammation, driving the progression and severity of the disease (Lai et al. 2023). The inflammatory burden in UC is substantial and a primary focus of current therapeutic strategies, a characteristic shared with other inflammatory conditions of the intestine (Cai et al. 2021).

At the cellular and molecular level, the pathogenesis of UC involves a cascade of events orchestrated by various signaling pathways and effector molecules. Key among these are growth factors crucial for tissue repair and angiogenesis, cytokines mediating inflammatory responses, and pathways regulating cell survival and death. Understanding the dysregulation of these elements in UC is vital for developing effective therapeutic interventions (Ning et al. 2024; Vaidyanathan 2021).

Growth factors like Vascular Endothelial Growth Factor (VEGF) and Epidermal Growth Factor (EGF) play complex roles in the context of UC. VEGF is a primary regulator of angiogenesis and vascular permeability. While essential for tissue repair by facilitating blood supply, its excessive upregulation in inflamed UC mucosa contributes to increased vascular permeability and edema, potentially exacerbating inflammation (Liashev et al. 2024; Zhu et al. 2021). Studies have shown that modulating VEGF signaling can impact the severity of colitis (Zoroddu et al. 2025). EGF, on the other hand, is a potent stimulus for epithelial cell proliferation, differentiation, and migration. Acting via its receptor, EGFR, EGF is critical for maintaining the integrity of the intestinal epithelial barrier and promoting the regeneration of damaged mucosa (Wang et al. 2021). Downregulation of EGF signaling in active UC is often associated with impaired mucosal healing (Villablanca et al. 2022). The intricate balance and interplay between VEGF and EGF are crucial for effective mucosal repair and resolution of inflammation in UC (Pompili et al. 2021).

Intracellular signaling pathways integrate diverse extracellular cues to orchestrate cellular responses in the gut. The Phosphoinositide 3‐Kinase/AKT (PI3K/AKT) pathway is a central signaling hub involved in crucial cellular processes including cell proliferation, survival, angiogenesis, and metabolism (Tarnawski and Ahluwalia 2021). Dysregulation of the PI3K/AKT pathway is frequently observed in the inflamed intestinal tissue of UC patients and experimental models, contributing significantly to the disease's pathogenesis (Jalil et al. 2023; Zhang et al. 2024). Both VEGF and EGF are known to activate the PI3K/AKT pathway downstream of their respective receptors, linking growth factor signaling to key cellular functions relevant to UC, such as epithelial repair (via EGF/EGFR/PI3K/AKT) and angiogenesis/inflammation (via VEGF/VEGFR/PI3K/AKT) (Chen et al. 2022; Stefani et al. 2021). Modulating the activity of this pathway has been proposed as a promising therapeutic strategy for UC.

Beyond growth factor and signaling pathway dysregulation, oxidative stress and aberrant apoptosis significantly contribute to the inflammatory cycle and tissue damage in UC. Oxidative stress results from an imbalance between the production of reactive oxygen species (ROS) and the capacity of the body's antioxidant defense systems (Total Oxidant Capacity—TOC, Total Antioxidant Capacity—TAC). The inflamed mucosa in UC is characterized by excessive ROS production, leading to oxidative damage to lipids, proteins, and DNA, which in turn fuels inflammation and impairs tissue repair (Arda‐Pirincci and Aykol‐Celik 2020; Zhang et al. 2021). Simultaneously, increased apoptosis, or programmed cell death, particularly among intestinal epithelial cells, disrupts the mucosal barrier integrity and contributes to the inflammatory burden (Wan et al. 2022). The delicate balance between pro‐apoptotic proteins (like Bax) and anti‐apoptotic proteins (like Bcl‐2), the release of Cytochrome c from mitochondria, and the activation of caspases (like Caspase‐9 and Caspase‐3) govern the apoptotic cascade (Ozal‐Coskun et al. 2024).

Given the multifactorial nature of UC, therapeutic strategies often aim to target multiple pathological processes. In recent years, approaches leveraging the potential of microorganisms and their components have gained significant attention. Probiotics, defined as live microorganisms that confer a health benefit on the host when administered in adequate amounts, have been explored for their ability to modulate the gut microbiota, enhance barrier function, and regulate immune responses in IBD and UC (Filidou and Kolios 2021; Lam et al. 2007). Specific probiotic strains, including various Lactobacillus species, have shown promise in alleviating symptoms and promoting remission in some UC patients (Côco et al. 2025; Jadhav et al. 2023). However, concerns regarding the viability of live probiotics, challenges in standardization, and potential risks in immunocompromised patients limit their widespread clinical application (Liang et al. 2024). This has led to increased interest in non‐viable microbial preparations, namely paraprobiotics and postbiotics. Paraprobiotics consist of inactivated microbial cells or their crude extracts, while postbiotics are soluble factors secreted by probiotics or released upon lysis (Martyniak et al. 2021). These non‐viable forms offer several advantages over live probiotics, including enhanced safety, longer shelf life, and ease of standardization and storage (Teame et al. 2020). Importantly, accumulating evidence suggests that paraprobiotics and postbiotics retain many of the beneficial properties of their live counterparts, and in some cases, may even exhibit superior or distinct therapeutic effects (Song et al. 2023; Teame et al. 2020).

The concept of utilizing biotics (probiotics, paraprobiotics, and postbiotics) to target UC has been investigated in numerous preclinical and clinical studies (Saedi et al. 2025; Štofilová et al. 2022). Research has demonstrated that these preparations can impact several key aspects of UC pathogenesis (Martyniak et al. 2021). Studies have shown their ability to modulate gut microbiota composition and function, enhance the intestinal epithelial barrier function, and regulate immune responses by modulating cytokine production, often reducing pro‐inflammatory cytokines like TNF‐α and IL‐6 while increasing anti‐inflammatory IL‐10 (Avci et al. 2024; Lee et al. 2024; Liu et al. 2024; Song et al. 2023; Teame et al. 2020). Furthermore, biotics have been shown to influence growth factor expression, such as increasing VEGF to promote vascularization or modulating EGF signaling to enhance epithelial repair (Chen et al. 2013; Kye et al. 2022; Lee et al. 2024; Zhang et al. 2015). Their capacity to reduce oxidative stress and mitigate oxidative damage is also well‐documented (Arda‐Pirincci and Aykol‐Celik 2020; Liu et al. 2024; Ozal‐Coskun et al. 2024; Ramani et al. 2023). Moreover, studies suggest that biotics can exert anti‐apoptotic effects, contributing to the preservation of intestinal cell survival and barrier integrity, and can modulate key signaling pathways like PI3K/AKT, linking their effects on growth factors, inflammation, and cell survival (Gao et al. 2023; Manna et al. 2024; Xu et al. 2022). The promising role of postbiotics, including those used in combination with approaches like fecal microbiota transplantation, is also being explored for their favorable safety profiles and efficacy in managing relapsing diseases like UC (Lê et al. 2022; Wang et al. 2021). These diverse findings underscore the potential of targeting multiple pathways with biotic interventions.

Given the marked inflammatory burden in UC, the search for paraprobiotics is of great interest as a therapeutic approach. Paraprobiotics, defined as non‐viable microbial cells, are obtained by various inactivation methods applied to live probiotics, including heat treatment, ultrasound, or UV‐C irradiation (Teame et al. 2020). The specific inactivation method used can significantly alter the properties of the paraprobiotic by affecting the integrity of cell surface molecules and the release of intracellular components. This difference in composition may in turn affect the bioactivity and therapeutic efficacy of the paraprobiotic, leading to method‐dependent differences in its immunomodulatory and tissue repair‐promoting capacities (Gholian et al. 2024). While the specific mechanisms by which UV‐C inactivation affects the retention or alteration of key surface molecules and intracellular components are the subject of ongoing research, the resulting paraprobiotic preparation represents a promising candidate for immunomodulation and tissue repair in UC (Kang et al. 2025).

Some studies have demonstrated that *Lacticaseibacillus casei* strains can reduce the severity of inflammation (Li et al. 2024; Wu et al. 2021). Given that UC is characterized by severe inflammatory responses and increased intestinal permeability, the use of live probiotics may pose certain safety concerns (Štofilová et al. 2022). In this context, the inactivated paraprobiotic form of *L. casei* is considered a potentially safe and effective therapeutic alternative, as it may modulate the inflammatory response while minimizing the risks associated with live probiotic administration. Based on this comprehensive background and the existing knowledge gap, the current study was designed to investigate the therapeutic effects of *L. casei* 39 paraprobiotic, prepared by UV‐C method, in an acetic acid‐induced rat model of UC. The primary aim was to evaluate its impact on macroscopic clinical indicators of colitis severity (DAI, body weight) and to elucidate its potential mechanisms of action by examining its effects on: (1) the expression of growth factors VEGF and EGF in colonic tissue; (2) systemic and local inflammatory cytokine profiles (TNF‐α, IL‐6, IL‐10); (3) the balance of total oxidants and antioxidants in colonic tissue; (4) the activation state of the central PI3K/AKT signaling pathway; and (5) the expression of key apoptotic markers (Bax, Bcl‐2, Caspase‐9, Caspase‐3, Cytochrome c). By assessing the multifaceted effects of *L. casei* 39 paraprobiotic on these interconnected pathways, this research seeks to provide critical insights into its therapeutic potential and contribute to the development of novel and effective strategies for UC management.

## 2 Materials and Methods

### 2.1 Materials

The probiotic strain *L. casei* 39 was sourced from Centro Sperimentale Del Latte, located in Zelo Buon Persico, Italy. All the chemicals and reagents utilized for the analyses were of analytical quality.

### 2.2 Paraprobiotic Preparation

*Lacticaseibacillus casei* 39 was weighed to reach a concentration of 10^8^ CFU/mL according to the manufacturer's recommendations and incubated in sterilized MRS broth medium in tightly sealed culture flasks at 37°C for 24 h (Akan et al. 2025). To determine probiotic growth, serial dilutions of the sample were prepared under aseptic conditions, spread on MRS agar plates, and incubated anaerobically at 37°C for 48–72 h using Anaerocult A (Merck, Darmstadt, Germany) (Terzaghi and Sandine 1975). When *L. casei* 39 reached a concentration of 10^8^ CFU/mL, the bacterial cells were centrifuged at 7000×*g* for 10 min at 4°C using a 50 mL Falcon tube. The supernatant was discarded, and the resulting bacterial pellet (probiotics to be inactivated) was washed three times with sterile phosphate buffered saline (PBS, pH 7.4) (Akan et al. 2025).

The bacterial suspension was spread evenly in a 1 mm thick layer on a sterile petri dish and irradiated with UV‐C light (UV lamp model EE4066LP UV solutionz, Kerikeri, New Zealand) at a wavelength of 254 nm with an output power of 72 W (Tan et al. 2020). The distance between the UV source and the sample was set to 16 cm. A total UV dose of 10,000 mJ/cm^2^ was applied to ensure effective bacterial inactivation. This dose was delivered in four consecutive treatments of 2500 mJ/cm^2^ each, with gentle mixing between treatments to maintain sample homogeneity. Following UV‐C treatment, the viability of probiotic cells was assessed on MRS agar, and no colony formation was observed, confirming complete inactivation. The washed pellet was resuspended in PBS for use in experiments (Almada et al. 2021).

### 2.3 Experimental Design and Animal Treatment

Female Wistar albino rats, weighing 250–300 g and aged 8–10 weeks, were used in this study. The rats were housed in a controlled laboratory environment with specific conditions (22°C ± 2°C temperature, 50%–60% humidity, 12‐h alternating light–dark cycle) with free access to regular rat chow and water. Before initiating the experiment (designated as Day 0), the animals underwent a 7‐day acclimatization period (Phase 1). On Day 7, UC was induced by rectal administration of 1 mL of 5% acetic acid solution under ketamine‐xylazine anesthesia (75 mg/kg −10 mg/kg) in all groups except the Control group (Phase 2, 1 day) (Abdel‐Daim et al. 2015). Treatments (oral gavage of 2 mL volume) were administered daily for 6 days (Phase 3), starting 1 day after colitis induction on Day 7. The rats were randomly divided into four groups (*n* = 10/group) as depicted in Table 1: Control Group (C; received PBS), UC Group (UC; received acetic acid + PBS), UC + Probiotic Group (UC‐PRO; received acetic acid + live *L. casei* 39 probiotic at 10^8^ CFU/mL), and UC + Paraprobiotic Group (UC‐PARA; received acetic acid + *L. casei* 39 paraprobiotic at 10^8^ CFU/mL) (Table 1). Sacrifice was performed after the 6‐day treatment period. All experimental protocols were carried out with the approval of the Animal Ethics Committee of Aydin Adnan Menderes University (Ethics Committee approval number: 64583101/2024/128, date: 05/12/2024).

**TABLE 1:** Experimental design of the study.

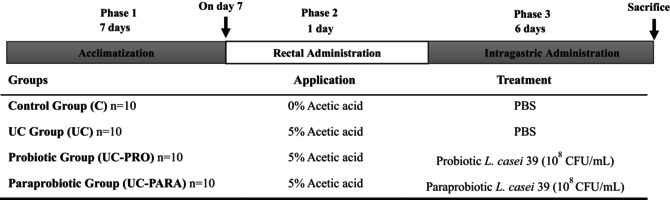

Wistar albino rats were divided into four experimental groups. Phase‐1 corresponded to a 7‐day acclimatization period. Phase‐2: On the following day, UC was induced by rectal administration of acetic acid in all groups except the control (C). Phase‐3: Treatments were administered via intragastric administration for 6 days after Phase‐2. The control group received 2 mL of PBS without acetic acid induction. The ulcerative colitis (UC) group received acetic acid and 2 mL of PBS. The probiotic (UC‐PRO) group received acetic acid and 2 mL of *L. casei* 39 probiotic. The paraprobiotic (UC‐PARA) group received acetic acid and 2 mL of *L. casei* 39 paraprobiotic.

### 2.4 Clinical Assessment of Colitis Severity

Throughout the 6‐day treatment period, body weights, stool consistency, and the presence of blood in the stool were monitored daily to assess colitis severity. Body weight change was calculated as a percentage relative to the initial weight recorded on Day 7. The disease activity index (DAI) was scored daily for each animal based on a composite scoring system that typically includes parameters such as weight loss, stool consistency, and gross bleeding (scores ranging from 0 for normal to 4 for severe). The specific scoring criteria followed a validated protocol for experimental colitis models (Shahid et al. 2022).

### 2.5 Sample Collection

Following the completion of the treatment protocol, the rats underwent deep sedation via an intraperitoneal injection consisting of ketamine (75 mg/kg) and xylazine (10 mg/kg). Blood was obtained from the sedated animals through cardiac puncture and collected in EDTA tubes. The colon was surgically removed, cut along its length, rinsed with PBS, and divided into two portions. One segment was employed for RNA isolation and subsequent quantitative real‐time polymerase chain reaction (qRT‐PCR) analysis, while the other was utilized to create tissue homogenates for Western blot analysis and ELISA procedures.

### 2.6 Tissue Homogenate Preparation

Samples of colon tissue were processed on ice using a homogenizer in a chilled protein extraction solution. This solution consisted of radio‐immunoprecipitation assay (RIPA, Sigma Aldrich R0278) buffer combined with a mixture of protease and phosphatase inhibitors (IKA, T 25 digital ultra‐turrax). The homogenization process was carried out for 1 min until the tissue was completely broken down. Following homogenization, the samples underwent centrifugation at 14,000 rpm for 20 min, maintained at 4°C. The resulting supernatants were extracted, and their protein content was quantified using the BCA protein assay kit (E‐BC‐K318‐M, Elabscience, China). Until further analysis, the prepared samples were kept in storage at −80°C.

### 2.7 ELISA for Systemic Inflammation Cytokines Measurement

Plasma samples obtained from blood were maintained at −80°C pending analysis. Subsequently, the quantities of TNF‐α, IL‐6, and IL‐10 were assessed using commercially procured ELISA kits (Rat TNF‐alpha ELISA Kit, Abcam; Rat IL‐6 ELISA Kit, Abcam, Rat IL‐10 ELISA Kit, Abcam, UK), adhering strictly to the manufacturer's specifications. Plasma samples underwent dilution as stipulated in the kit protocol prior to analysis, with each sample examined in duplicate. Absorbance measurements were recorded at 450 nm employing a Thermo Scientific Multiskan Spectrum microplate reader. The concentrations of TNF‐α, IL‐6, and IL‐10 were computed using a standard curve.

### 2.8 Determination of TOC and TAC Levels

Commercial kits from Rel Assay Diagnostics (Gaziantep, Turkey) were utilized to evaluate colon tissue specimens for TOC and TAC. The analyses were conducted in accordance with the manufacturer's guidelines, employing spectrophotometric techniques for both assays. TOC levels were measured based on the principle that oxidants in the samples oxidize the chromogen o‐dianisidine to form a colored product. The intensity of the color change, measured spectrophotometrically at 530 nm using a microplate reader (Thermo Scientific Multiskan Spectrum, USA), is proportional to the total oxidant concentration. TOC levels were expressed as μmol H_2_O_2_ equivalent/L.

TAC levels were determined using a method based on the ability of anti‐oxidants in the samples to reduce ABTS (2,2′‐azino‐bis (3‐ethylbenzothiazoline‐6‐sulfonic acid)) radical, causing a color change. The absorbance was measured spectrophotometrically at 660 nm. TAC levels were expressed as mmol Trolox equivalent/L.

### 2.9 RNA Isolation and cDNA Synthesis

Colon tissue samples underwent RNA extraction using a commercial kit (RNeasy Plus Mini Kit Cat. No. 74134), following the manufacturer's protocol. A spectrophotometer (NanoDrop, Thermo Scientific, USA) was employed to assess RNA quality and quantity. Samples exhibiting a 260/280 nm absorbance ratio between 1.8 and 2.0 were considered appropriate for subsequent examination.

cDNA synthesis was performed using a high‐capacity cDNA reverse transcription kit (High‐Capacity cDNA Reverse Transcription Kit, Applied Biosystems Cat No: 4374967, USA) according to the manufacturer's instructions. For each reaction, 1 μg of total RNA was used. The reverse transcription reaction was performed using a thermal cycler (C1000 Touch PCR thermal cycler, Biorad, USA) with the following program: 10 min at 25°C, 120 min at 37°C, and 5 min at 85°C. The synthesized cDNAs were stored at −20°C.

### 2.10 qRT‐PCR for VEGF and EGF Gene Expression Analysis

The expression levels of VEGF and EGF genes were examined using qRT‐PCR. This technique employed a SYBR Green‐based PCR master mix, specifically the PowerUp SYBR Green Master Mix from Applied Biosystems, along with gene‐specific primers. Table 2 listed the primer sequences used in this study.

**TABLE 2:** Primers for qRT‐PCR.

Primers	Sequence
VEGF	F: 5′ ATCATGCGGATCAAACCTCACC 3′ R: 5′ GGTCTGCATTCACATCTGCTATGC 3′
EGF	F: 5′ CCACGGTTACATTCACTCC 3′ R: 5′ GCTATCCAAATCGCCTTC 3′
GAPDH	F: 5′ AGGTCGGTGTGAACGGATTTG 3′ R: 5′ GGGGTCGTTGATGGCAACA 3′

qRT‐PCR reactions were performed using a qRT‐PCR instrument (Applied Biosystems StepOnePlus Real‐Time PCR System, USA) with the following program: 10 min at 95°C (initial denaturation), followed by 40 cycles of 15 s at 95°C (denaturation) and 1 min at 60°C (annealing and extension). Each sample was run in triplicate.

The 2^−ΔΔCt^ method was employed to determine the comparative expression levels of VEGF and EGF genes. The GAPDH gene served as the internal control for this analysis (Abdel‐Daim et al. 2015).

### 2.11 Western Blot Analysis of Local Inflammation Proteins, Apoptosis and PI3K/AKT Signaling Pathway Proteins

Proteins from tissue homogenate (30 μg/well) were initially separated using 10%–12% SDS‐PAGE. Subsequently, these proteins were transferred onto PVDF membranes through an electrophoretic process (Towbin et al. 1979). These membranes underwent a blocking process with 5% non‐fat dry milk in TBST for 1 h at room temperature. Subsequently, the membranes were incubated with primary antibodies at 4°C overnight. The antibodies included anti‐PI3K (1:1000 dilution, Cell Signaling Technology, USA), anti‐p‐PI3K (1:1000 dilution, Sigma‐Aldrich, Germany), anti‐AKT (1:1000 dilution, Cell Signaling Technology, USA), anti‐p‐AKT (Ser473) (1:1000 dilution, Cell Signaling Technology, USA), caspase‐3 (1:500 dilution, Cell Signaling Technology, USA), Bax (1:500 dilution, Cell Signaling Technology, USA), Bcl‐2 (1:500 dilution, Cell Signaling Technology, USA), cytochrome‐c (cyt‐c) (1:750 dilution, Cell Signaling Technology, USA), caspase‐9 (1:500 dilution, Cell Signaling Technology, USA), IL‐6 (1:750 dilution, Invitrogen, CA, USA), IL‐10 (1:750 dilution, Invitrogen, CA, USA), TNF‐α (1:750 dilution, eBioscience, CA, USA) and anti‐β‐actin (1:5000 dilution, Abcam, UK). After washing, the membranes were exposed to HRP‐linked secondary antibodies (1:2000 dilution, Cell Signaling Technology, USA) for 60 min at room temperature. Chemiluminescent ECL substrates (SuperSignal West Pico PLUS Chemiluminescent Substrate, Thermo Scientific, USA) were utilized to detect protein bands. These bands were captured and documented using a G:Box Imaging System (Syngene, UK). Band intensity analysis was performed using ImageJ software. The expression levels of PI3K, p‐PI3K, AKT, p‐AKT, Bax, Bcl‐2, caspase‐3, caspase‐9, IL‐6, IL‐10, and TNF‐α were standardized against β‐actin, which functioned as the reference protein.

### 2.12 Statistical Analysis

The findings were expressed as mean values with their standard deviations (SD). To evaluate disparities among groups, one‐way analysis of variance (ANOVA) was performed, followed by Tukey's multiple comparison test using GraphPad Prism 9 software. A *p*‐value < 0.05 was considered statistically significant.

## 3 Results

### 3.1 Amelioration of Clinical Symptoms in UC Rats by *L. casei* 39 Paraprobiotic

**FIGURE 1:**
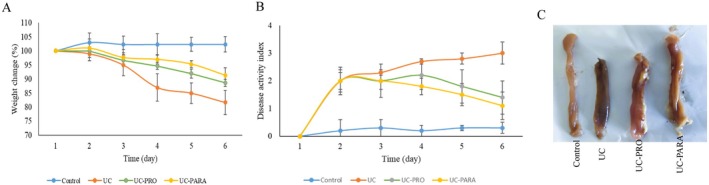
Effects of *Lacticaseibacillus casei* 39 probiotic and paraprobiotic on clinical indicators of experimental UC in rats. (A) Percentage body weight changes relative to the initial weight on Day 1 of treatment. (B) Disease Activity Index (DAI) score is recorded daily. (C) Representative macroscopic images of excised colon tissues from each experimental group at the end of the study. Groups include Control (received PBS), Ulcerative Colitis (UC; received acetic acid + PBS), UC + Probiotic (UC‐PRO; received acetic acid + probiotic *L. casei* 39), and UC + Paraprobiotic (UC‐PARA; received acetic acid + *L. casei* 39 paraprobiotic). Data in panels A and B are presented as mean ± standard deviation (SD) (*n* = 10/group).

Clinical indicators of colitis severity, including body weight change and Disease Activity Index (DAI), were monitored daily for 6 days following UC induction and the initiation of treatments. As shown in Figure 1A, rats in the Control group maintained stable body weight throughout the study period. In contrast, the UC group exhibited significant body weight loss starting from Day 2 and progressively worsening until Day 6. Both the UC‐PRO and UC‐PARA groups showed attenuated weight loss compared to the untreated UC group, indicating a protective effect of both probiotic and paraprobiotic treatments on this clinical parameter.

The DAI score, a composite measure reflecting the severity of colitis symptoms, paralleled the weight change observations (Figure 1B). The control group maintained a DAI score near 0. The UC group developed severe colitis, characterized by a rapid increase in DAI score from Day 2 onwards, reaching a peak by Day 6. Both UC‐PRO and UC‐PARA treatments significantly reduced the increase in DAI score compared to the UC group, indicating a notable amelioration of clinical colitis symptoms. The DAI scores in the treated groups were consistently lower than in the untreated UC group throughout the observation period.

### 3.2 Effects of *L. casei* 39 Paraprobiotic on Systemic and Local Inflammatory Cytokine Levels

**FIGURE 2:**
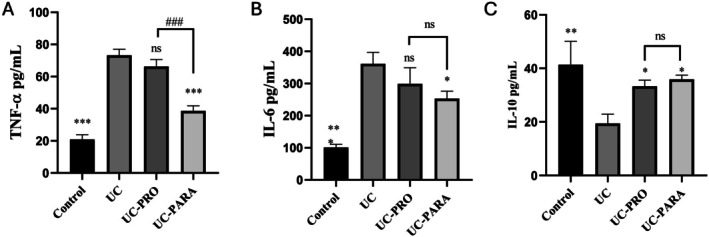
Impact of *Lacticaseibacillus casei* 39 paraprobiotic on TNF‐α, IL‐6, and IL‐10 concentrations in plasma. (A) TNF‐α concentrations. (B) IL‐6 concentrations. (C) IL‐10 concentrations. Data presented mean values with SD (*n* = 10 for each group). ***p < 0.001, **p < 0.01, *p < 0.05 versus UC. ###*p* < 0.001 indicates statistical significance between UC‐PRO and UC‐PARA groups. ns, non significant.

To assess the effects of *L. casei* 39 paraprobiotic on systemic and local (colonic) inflammation, the concentrations of inflammatory markers in plasma were quantified using enzyme‐linked immunosorbent assay (ELISA). The analysis focused on three specific cytokines: the pro‐inflammatory markers TNF‐α and IL‐6, and the anti‐inflammatory cytokine IL‐10. Figure 2A illustrates the findings, demonstrating that TNF‐α levels were significantly higher in the UC group compared to the control group (*p* < 0.001). Although the administration of probiotics did not significantly lower TNF‐α levels (*p* > 0.05), the use of paraprobiotics resulted in a marked reduction of TNF‐α levels in comparison to the UC group (*p* < 0.001).

Likewise, the UC group demonstrated notably elevated concentrations of IL‐6 when compared to the control group (*p* < 0.001) (Figure 2B). Once more, the administration of probiotics did not yield a significant impact on IL‐6 concentrations (*p* > 0.05). Conversely, administering paraprobiotics led to a significant reduction in IL‐6 concentrations (*p* < 0.05).

In contrast, IL‐10 levels significantly decreased in the UC group compared to the control group (*p* < 0.01) (Figure 2C). Compared to the UC group, the levels of IL‐10 were significantly elevated in both the probiotic and paraprobiotic treatment groups (*p* < 0.05).

**FIGURE 3:**
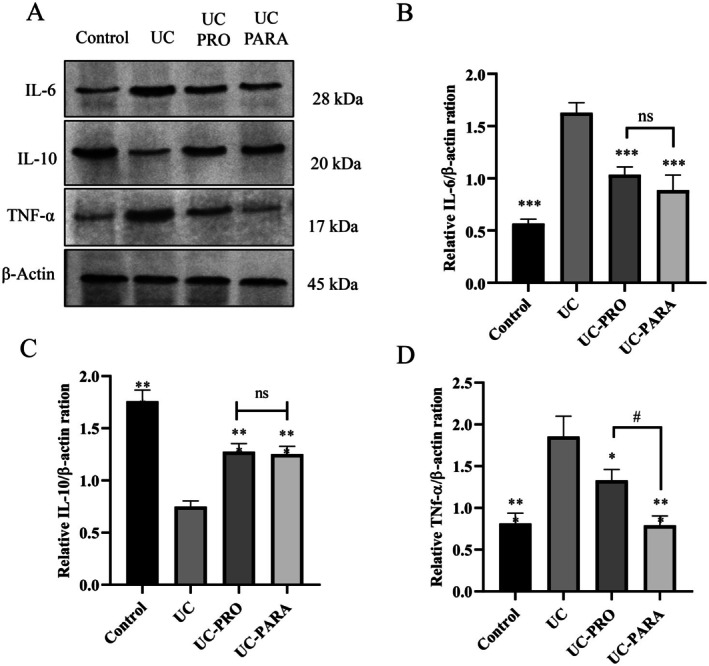
Effects of *Lacticaseibacillus casei* 39 paraprobiotic on inflammatory cytokine protein expression in colon tissue of UC rats. (A) Representative Western blot images showing protein levels of IL‐6, IL‐10, TNF‐α, and the loading control β‐Actin in colon tissue homogenates from each experimental group. (B) Quantitative analysis of relative IL‐6 protein expression normalized to β‐Actin. (C) Quantitative analysis of relative IL‐10 protein expression normalized to β‐Actin. (D) Quantitative analysis of relative TNF‐α protein expression normalized to β‐Actin. Groups are Control, Ulcerative Colitis (UC), UC + Probiotic ( *L. casei* 39) (UC‐PRO), and UC + Paraprobiotic ( *L. casei* 39) (UC‐PARA). Data in panels B, C, and D are presented as mean ± SD (*n* = 3 pooled samples/group). Statistical significance: *p < 0.05, **p < 0.01, ***p < 0.001 compared to the UC group. #*p* < 0.05 compared to the UC‐PRO group. ns, not significant.

Western blot analysis was performed on colon tissue homogenates to quantify the protein expression levels of key cytokines (IL‐6, IL‐10, and TNF‐α), aiming to evaluate the local inflammatory response to the *L. casei* 39 paraprobiotic. As illustrated in Figure 3A, the representative Western blot images are presented, while Figure 3B–D demonstrate the quantitative analysis that has been normalized to β‐actin.

The relative expression of the IL‐6 protein (Figure 3B) was found to be significantly increased in the UC group in comparison with the Control group (*p* < 0.001). The investigation revealed that the administration of probiotics (UC‐PRO) did not result in a substantial reduction in IL‐6 levels when compared to the UC group (*p* > 0.05). However, the administration of paraprobiotic treatment (UC‐PARA) resulted in a substantial reduction in IL‐6 expression in comparison with the UC group (*p* < 0.001). In contrast, relative IL‐10 protein expression (Figure 3C) was significantly decreased in the UC group compared to the Control group (*p* < 0.001). The present study demonstrated that both probiotic (UC‐PRO) and paraprobiotic (UC‐PARA) treatments resulted in a significant increase in IL‐10 expression in comparison with the UC group (*p* < 0.001 for both).

The relative TNF‐α protein expression levels (Figure 3D) were found to be significantly elevated in the UC group in comparison to the Control group (*p* < 0.001). The administration of probiotics (UC‐PRO) resulted in a significant reduction in TNF‐α levels in comparison with the UC group (*p* < 0.05). Paraprobiotic treatment (UC‐PARA) led to a more pronounced and statistically significant decrease (*p* < 0.001), and TNF‐α expression was significantly lower in the paraprobiotic group compared to the probiotic group (*p* < 0.05).

### 3.3 Effect of *L. casei* 39 Paraprobiotic on Colonic Total Oxidant and Antioxidant Levels

**FIGURE 4:**
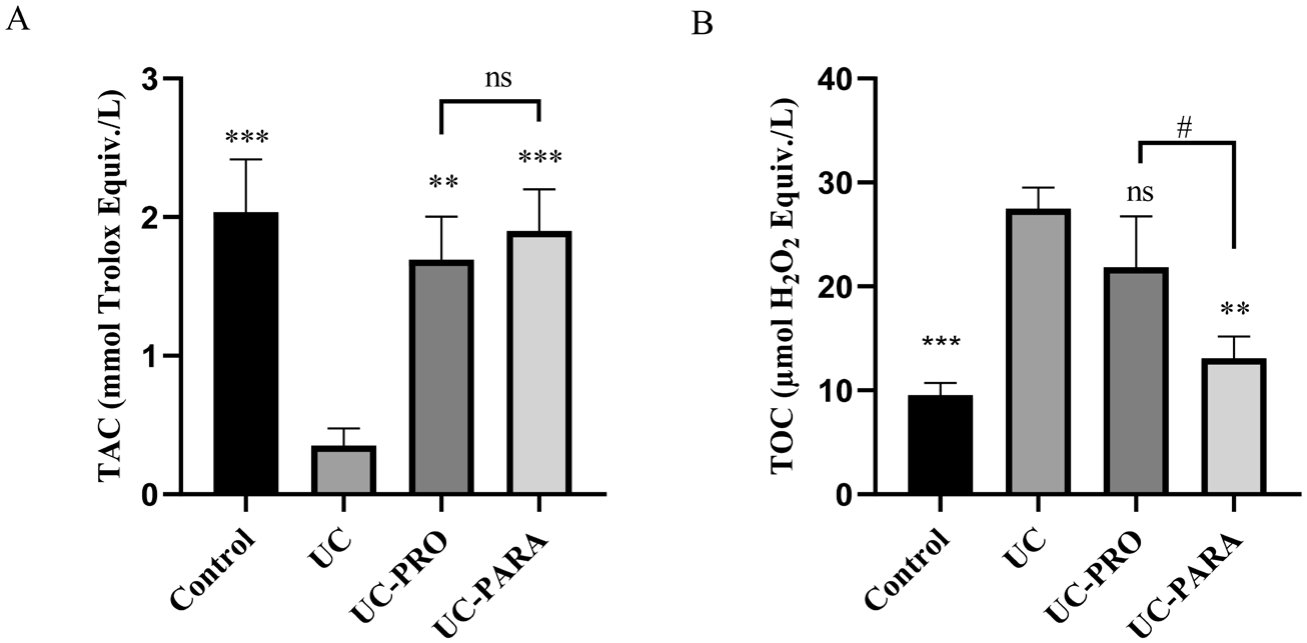
(A) Effect of *Lacticaseibacillus casei* 39 paraprobiotic on colonic TOC. (B) Effect of *L. casei* 39 paraprobiotic on colonic TAC values presented as mean ± SD. ***p < 0.001, **p < 0.01 versus UC. #*p* < 0.05 indicates statistical significance between UC‐PRO and UC‐PARA groups. ns, non significant (*n* = 10 for each group).

TOC and TAC levels were analyzed on rat colon tissues. Figure 4A shows that there was a significant increase (2.88‐fold) in TOC in the UC group compared to the control (*p* < 0.001). Interestingly, it did not cause significant changes in TOC levels in the PRO group (*p* > 0.05). On the contrary, paraprobiotic treatment led to a significant decrease in TOC compared to the UC group, approaching control group levels. A 2.10‐fold decrease in TOC level was observed in the PARA group compared to the UC group (*p* < 0.01). As shown in Figure 4B, TAC was significantly lower in the UC group than in control. An approximately 5.82‐fold decrease was found (*p* < 0.001). Compared to the UC group, significant increases in TAC levels were detected after administration of both probiotic and paraprobiotic. In the PRO group, there was a 4.83‐fold increase (*p* < 0.01), while in the PARA group this increase was 5.43‐fold (*p* < 0.001).

### 3.4 Effects of *L. casei* 39 Paraprobiotic on VEGF and EGF Gene Expression

**FIGURE 5:**
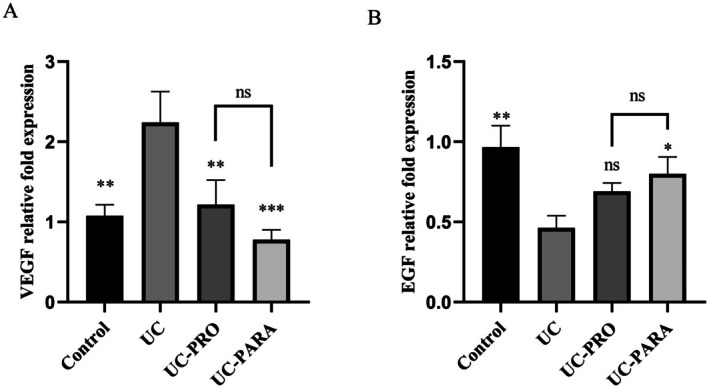
Effects of *Lacticaseibacillus casei* 39 paraprobiotic on colonic VEGF and EGF mRNA expression. (A) VEGF relative fold expression. (B) EGF relative fold expression. Results are illustrated as mean ± SD (*n* = 10/group). ***p < 0.001 **p < 0.01 *p < 0.05 versus UC. ns, non significant.

To investigate the effects of *L. casei* 39 paraprobiotic on mucosal healing, the mRNA expression of VEGF and EGF was assessed in colon samples using qRT‐PCR. Figure 5A shows that VEGF expression increased 2.08‐fold in the UC group compared to the control group (*p* < 0.01). When probiotics were administered, VEGF expression decreased 1.84‐fold compared to the UC group (*p* > 0.01). In contrast, administration of paraprobiotics resulted in a more significant decrease in VEGF expression (2.87‐fold) compared to the UC group (*p* < 0.001).

Conversely, the UC group exhibited a significant decrease in EGF expression compared to the control group. EGF expression was decreased 2.08‐fold in the UC group compared to the control group (*p* < 0.01) (Figure 5B). Compared to the UC group, EGF expression decreased in the PRO group, but no statistically significant decrease was observed (*p* > 0.05). However, a 1.73‐fold increase in EGF expression was observed in the PARA group compared to the UC group (*p* < 0.05). Paraprobiotic treatment showed a more significant effect on EGF expression.

### 3.5 Effects of *L. casei* 39 Paraprobiotic on PI3K/AKT Pathway Activation

**FIGURE 6:**
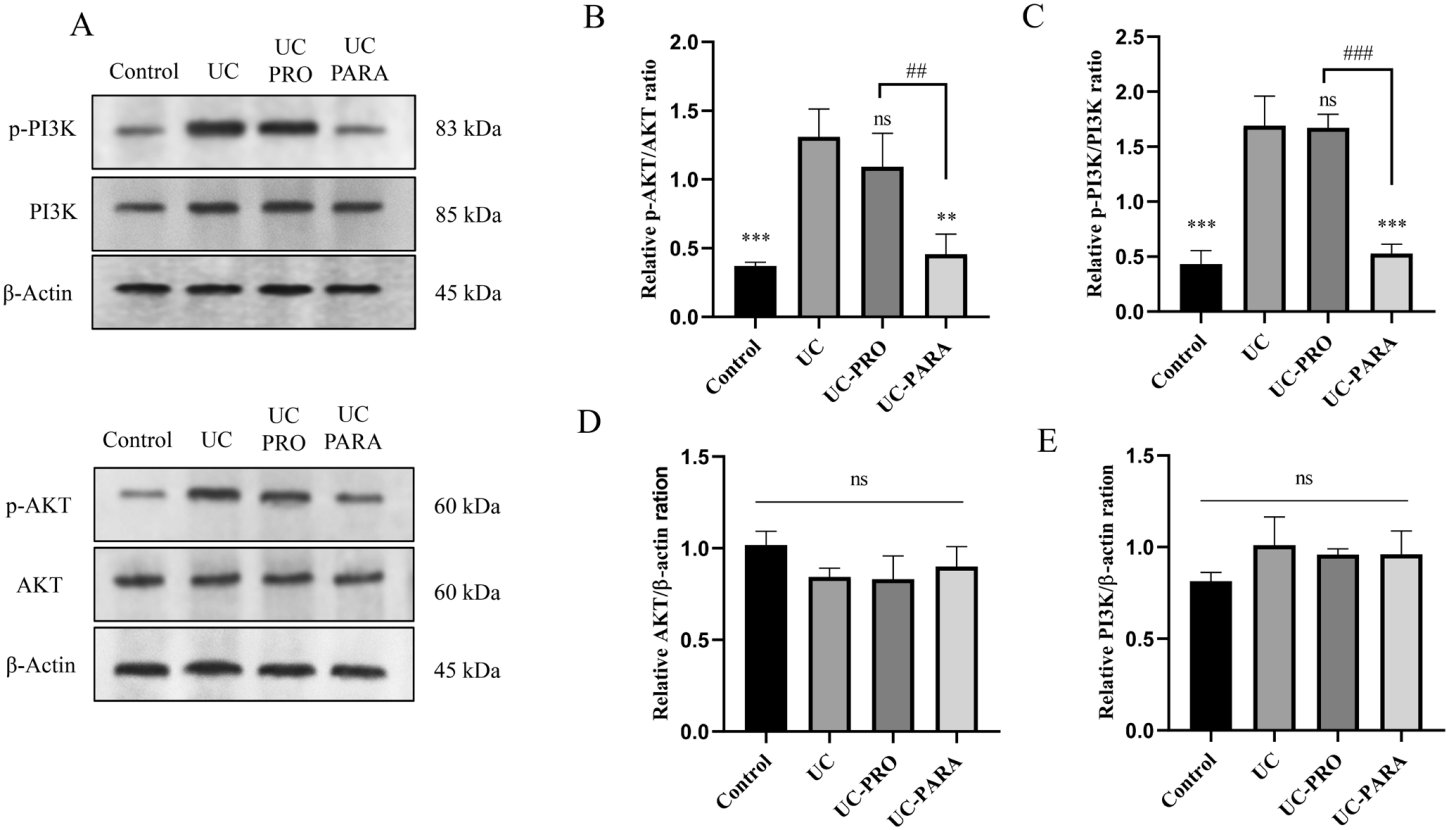
Effects of *Lacticaseibacillus casei* 39 paraprobiotic on PI3K/AKT pathway protein expression in colon tissue. (A) Western blot band images of p‐PI3K, PI3K, p‐AKT, AKT, and β‐Actin (B) Relative p‐AKT/AKT ratio. (C) Relative p‐PI3K/PI3K ratio (D) Relative AKT/β‐Actin ratio (E) Relative PI3K/β‐Actin ratio. Data are presented as mean ± SD (*n* = 10/group). ***p < 0.001, **p < 0.01 versus UC. ###*p* < 0.001, ##*p* < 0.01 indicates statistical significance between UC‐PRO and UC‐PARA groups. ns, non significant.

Western blot analysis was performed on colon tissue homogenates to evaluate whether the therapeutic effects of *L. casei* 39 paraprobiotic in UC were associated with alterations in the expression of key proteins within the PI3K/AKT signaling pathway. Figure 6A shows representative Western blot images for p‐PI3K, PI3K, p‐AKT, AKT, and the β‐actin loading control across the experimental groups. Figure 6B–E present the quantitative analysis of these protein levels.

The ratio of p‐AKT/AKT provides a measure of AKT activation. Figure 6B shows that the p‐AKT/AKT ratio was significantly increased in the UC group compared to the Control group (approximately 1.3‐fold relative ratio compared to Control, *p* < 0.001), indicating significant activation of AKT in colitis. Probiotic treatment (UC‐PRO) did not lead to a statistically significant reduction in the p‐AKT/AKT ratio compared to the UC group (*p* > 0.05). However, paraprobiotic treatment (UC‐PARA) significantly reduced the p‐AKT/AKT ratio compared to the UC group (approximately 0.4‐fold relative ratio compared to Control, *p* < 0.01). Similarly, the ratio of p‐PI3K/PI3K indicates PI3K activation. Figure 6C shows that the p‐PI3K/PI3K ratio was significantly increased in the UC group compared to the Control group (approximately 1.7‐fold relative ratio compared to Control, *p* < 0.001). Probiotic treatment (UC‐PRO) did not significantly reduce the p‐PI3K/PI3K ratio compared to the UC group (*p* > 0.05). Paraprobiotic treatment (UC‐PARA) significantly reduced the p‐PI3K/PI3K ratio compared to the UC group (approximately 0.5‐fold relative ratio compared to Control, *p* < 0.001).

Total protein levels of AKT and PI3K, normalized to the β‐actin loading control, were analyzed to assess whether the treatments affected the overall amount of these proteins. Figure 6D shows no statistically significant differences in total AKT expression (AKT/β‐actin ratio) among the Control, UC, UC‐PRO, and UC‐PARA groups (*p* > 0.05). Likewise, Figure 6E shows no statistically significant differences in total PI3K expression (PI3K/β‐actin ratio) among the groups (*p* > 0.05). This indicates that the observed effects were primarily on the phosphorylation (activation) state of PI3K and AKT, rather than on their total protein levels.

### 3.6 Effects of *L. casei* 39 Paraprobiotic on Apoptosis Markers in Colon Tissue

**FIGURE 7:**
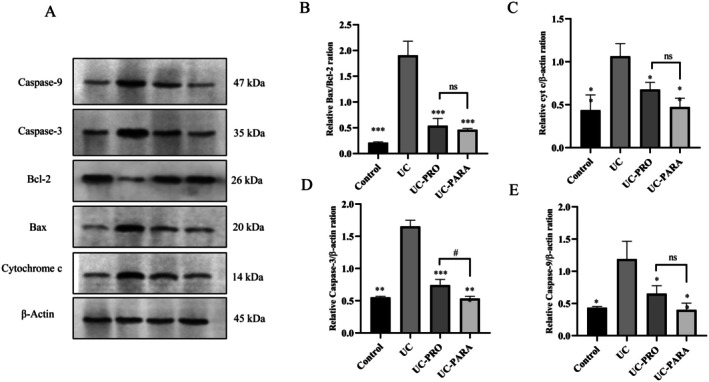
Effects of *Lacticaseibacillus casei* 39 Paraprobiotic on Apoptosis Marker Protein Expression in Colon Tissue of UC Rats. (A) Representative Western blot images for Caspase‐9, Caspase‐3, Bcl‐2, Bax, Cytochrome c, and β‐Actin proteins from the experimental groups. β‐Actin was used as a loading control. (B) Quantitative analysis of Bax/Bcl‐2 expression ratios. (C) Quantitative analysis of Cytochrome c expression levels normalized to β‐Actin. (D) Quantitative analysis of Caspase‐3 expression levels normalized to β‐Actin. (E) Quantitative analysis of Caspase‐9 expression levels normalized to β‐Actin. Data are presented as mean ± SD (*n* = 3 pooled samples/group). Statistical significance: ***p < 0.001, **p < 0.01, *p < 0.05 versus UC. ns, not significant. #*p* < 0.05 versus UC‐PRO.

Western blot analysis was conducted to investigate the effects of *L. casei* 39 paraprobiotic on the expression of major apoptotic and anti‐apoptotic markers in the colonic tissues of rats with UC. Figure 7A shows representative Western blot images for Caspase‐9, Caspase‐3, Bcl‐2, Bax, Cytochrome c (cyt c), and β‐actin across the experimental groups. Figure 7B–E present the quantitative analysis of these protein levels.

Apoptosis is often regulated by the balance between pro‐apoptotic (e.g., Bax) and anti‐apoptotic (e.g., Bcl‐2) proteins. Figure 7B shows that the ratio of Bax to Bcl‐2 expression was significantly increased in the UC group compared to the Control group (approximately 1.9‐fold relative ratio compared to Control, *p* < 0.001), indicating a shift towards pro‐apoptotic signaling. Both probiotic (UC‐PRO) and paraprobiotic (UC‐PARA) treatments significantly reduced the Bax/Bcl‐2 ratio compared to the UC group (approximately 0.5‐fold relative ratio compared to Control, *p* < 0.001 for both). No significant difference was observed between the UC‐PRO and UC‐PARA groups regarding this ratio (*p* > 0.05).

Cyt c release from the mitochondria into the cytoplasm is an early event in the intrinsic apoptotic pathway. Figure 7C shows that Cyt c expression was significantly increased in the UC group compared to the Control group (approximately 1.0‐fold relative expression compared to Control, *p* < 0.01). Both UC‐PRO and UC‐PARA treatments significantly reduced Cyt c expression compared to the UC group (approximately 0.7‐fold and 0.5‐fold relative expression compared to Control, *p* < 0.05 and *p* < 0.01, respectively). No significant difference was observed between the UC‐PRO and UC‐PARA groups (*p* > 0.05).

Caspase‐3 is a key executioner caspase in apoptosis. Figure 7D shows that Caspase‐3 expression was significantly increased in the UC group compared to the Control group (approximately 1.6‐fold relative expression compared to Control, *p* < 0.001). Probiotic treatment (UC‐PRO) led to a significant reduction in Caspase‐3 expression compared to the UC group (approximately 0.7‐fold relative expression compared to Control, *p* < 0.001). Paraprobiotic treatment (UC‐PARA) also significantly reduced Caspase‐3 expression compared to the UC group (approximately 0.5‐fold relative expression compared to Control, *p* < 0.001). Furthermore, Caspase‐3 expression was significantly lower in the UC‐PARA group compared to the UC‐PRO group (*p* < 0.05).

Caspase‐9 is an initiator caspase in the intrinsic apoptotic pathway. Figure 7E shows that Caspase‐9 expression was significantly increased in the UC group compared to the Control group (approximately 1.2‐fold relative expression compared to Control, *p* < 0.01). Probiotic treatment (UC‐PRO) significantly reduced Caspase‐9 expression compared to the UC group (approximately 0.7‐fold relative expression compared to Control, *p* < 0.05). Paraprobiotic treatment (UC‐PARA) also significantly reduced Caspase‐9 expression compared to the UC group (approximately 0.4‐fold relative expression compared to Control, *p* < 0.01). No significant difference was observed between the PRO and PARA groups (*p* > 0.05). Beta‐actin protein levels remained consistent across all experimental groups, confirming equal protein loading (Figure 7A).

## 4 Discussion

This study aimed to evaluate the therapeutic potential of *L. casei* 39 paraprobiotic, produced via UV‐C inactivation of probiotic *L. casei* 39, in a rat model of UC induced by acetic acid. Our findings reveal that this paraprobiotic significantly ameliorated key aspects of UC pathology, including improving macroscopic clinical indicators, modulating growth factor expression, reducing inflammation and oxidative stress, suppressing the activation of the PI3K/AKT signaling pathway, and mitigating apoptosis. These results provide comprehensive preclinical evidence supporting the utility of UV‐C‐inactivated *L. casei* 39 as a potential multi‐target therapeutic agent for UC.

The most immediate indicators of therapeutic efficacy in preclinical colitis models are improvements in clinical signs such as body weight loss and DAI. Our data demonstrated that rats treated with *L. casei* 39 paraprobiotic exhibited significantly attenuated weight loss and reduced DAI scores compared to the untreated UC group (Figure 1A,B). These findings are consistent with numerous studies showing that probiotic and paraprobiotic interventions can effectively ameliorate macroscopic symptoms in chemically induced colitis models. Liu et al. (2024) reported that paraprobiotics derived from *Limosilactobacillus fermentum* significantly reduced DAI and improved colon length in DSS‐induced colitis mice (Liu et al. 2024). Similarly, Kye et al. (2022) demonstrated that a *Lactobacillus acidophilus* paraprobiotic ameliorated DSS‐induced colitis, reflected in reduced clinical scores and histological damage (Kye et al. 2022). The comparable efficacy of the *L. casei* 39 paraprobiotic to the live probiotic in improving these clinical indicators (Figure 1A,B) is particularly promising, reinforcing the concept that non‐viable forms can offer therapeutic benefits without the inherent risks of live bacteria, making them potentially safer alternatives, especially for immunocompromised patients or those with severe UC (Song et al. 2023; Teame et al. 2020). The macroscopic visual improvements in colon morphology in the paraprobiotic group (Figure 1C) further support these clinical findings.

Inflammation plays a central role in the pathogenesis of UC, and is characterized by dysregulated cytokine production at both systemic and colonic levels (Segal et al. 2021). Our analysis of plasma cytokines (Figure 2) showed significantly elevated pro‐inflammatory TNF‐α and IL‐6 and reduced anti‐inflammatory IL‐10 in the untreated UC group. Paraprobiotic treatment effectively reversed these changes, significantly reducing plasma TNF‐α and IL‐6 and increasing IL‐10. At the local site of disease, Western blot analysis in colon tissue confirmed increased protein expression of pro‐inflammatory IL‐6 and TNF‐α and decreased IL‐10 in the UC group (Figure 3). Consistent with the systemic effects, paraprobiotic treatment significantly reduced local IL‐6 and TNF‐α protein expression and increased IL‐10 protein expression. The robust anti‐inflammatory capacity of *L. casei* 39 paraprobiotic, acting both systemically and locally, is a key finding. This aligns with the known immunomodulatory effects of paraprobiotics, which can interact with host immune cells to shift the cytokine balance towards an anti‐inflammatory state (Avci et al. 2024; Teame et al. 2020). Recent studies have provided direct evidence supporting this. Liu et al. (2024) demonstrated that *Limosilactobacillus fermentum* paraprobiotic alleviated intestinal inflammation by downregulating pro‐inflammatory cytokine expression, while Song et al. (2023) reported that *Lactiplantibacillus plantarum* paraprobiotic effectively attenuated pro‐inflammatory responses in a murine model of colitis. The observation that the paraprobiotic induced a significantly greater reduction in local TNF‐α protein expression compared to the live probiotic (Figure 3D) is particularly noteworthy, suggesting that the UV‐C‐inactivated form may possess distinct or enhanced local immunomodulatory properties for specific inflammatory mediators (Song et al. 2023). Oxidative stress, stemming from an imbalance between oxidants and antioxidants, is a major contributor to inflammation‐induced tissue damage in UC (Arda‐Pirincci and Aykol‐Celik 2020; Ozal‐Coskun et al. 2024; Zhang et al. 2021). Our results demonstrated a significant increase in colon TOC and a decrease in TAC in the untreated UC group (Figure 4A,B). Treatment with *L. casei* 39 paraprobiotic effectively mitigated this oxidative imbalance, significantly reducing TOC and increasing TAC levels. This suggests that the paraprobiotic possesses intrinsic antioxidant properties or enhances endogenous antioxidant defense mechanisms in the inflamed colon. Preclinical studies have shown that probiotics and paraprobiotics can reduce oxidative stress markers and improve antioxidant enzyme activities in colitis models (Liu et al. 2024; Ramani et al. 2023). Aydın et al. (2021) evaluated the antioxidant effects of postbiotics and paraprobiotics derived from lactic acid bacteria (Aydın et al. 2021). Liu et al. (2024) specifically demonstrated that *Limosilactobacillus fermentum* paraprobiotic could improve intestinal barrier function and reduce oxidative stress in mice with UC (Liu et al. 2024). Our findings corroborate these studies and highlight the potential of *L. casei* 39 paraprobiotic to protect against oxidative damage in the context of UC.

VEGF and EGF are growth factors with critical, albeit complex, roles in UC pathogenesis and mucosal healing. Elevated VEGF contributes to vascular changes and inflammation (Kanazawa et al. 2001) while EGF is essential for epithelial repair (Cong et al. 2024). Our study revealed increased VEGF mRNA and decreased EGF mRNA expression in the colon of UC rats (Figure 5A,B), consistent with impaired healing and inflammation. *L. casei* 39 paraprobiotic treatment favorably modulated this balance, significantly reducing VEGF mRNA and increasing EGF mRNA expression. This suggests a mechanism by which the paraprobiotic promotes both reduced inflammation (via potential anti‐angiogenic or reduced permeability effects through VEGF modulation) and enhanced epithelial regeneration (via increased EGF). The ability of biotics to influence growth factor expression is gaining recognition (Teame et al. 2020; Zawistowska‐Rojek and Tyski 2022). Kye et al. (2022) linked a *L. acidophilus* paraprobiotic to intestinal healing potentially through EGF modulation, and Lee et al. (2024) suggested paraprobiotics influenced immune responses and gut microbiota potentially through EGF pathways (Kye et al. 2022; Lee et al. 2024). Liu et al. (2024) also reported that paraprobiotics improve intestinal barrier, which is linked to growth factor balance. Our specific findings for *L. casei* 39 paraprobiotic demonstrate its capacity to create a growth factor environment conducive to mucosal repair.

The PI3K/AKT signaling pathway is a central mediator of cell survival, proliferation, inflammation, and angiogenesis, and its dysregulation is implicated in UC (Xu et al. 2022; Zhang et al. 2021). The Western blot analysis showed significant activation of the PI3K/AKT pathway in the colon of UC rats, as indicated by an increased ratio of phosphorylated (active) PI3K and AKT to their total forms (Figure 6B,C). Importantly, *L. casei* 39 paraprobiotic treatment significantly suppressed this activation. Inhibition of the PI3K/AKT pathway during acute inflammation may be beneficial by limiting pro‐inflammatory signaling and potentially promoting the removal of damaged cells (Xu et al. 2022; Zhang et al. 2021). Modulation of the PI3K/AKT pathway by biotics has been reported; Cong et al. (2024) showed that EGFR activation stimulates the pathway, and Song et al. (2023) reported that certain probiotic strains can modulate the PI3K/AKT pathway (Cong et al. 2024; Song et al. 2023). Zhang et al. (2024) showed that blocking the PI3K/AKT pathway alleviated colitis. Our findings position *L. casei* 39 paraprobiotic among the biotics capable of modulating the PI3K/AKT pathway, suggesting a potential mechanistic basis for its anti‐inflammatory and anti‐apoptotic effects. Notably, the marked suppression of PI3K/AKT activation by the paraprobiotic underscores the therapeutic potential of the inactivated form in UC.

Excessive apoptosis of intestinal epithelial cells is a pathological feature of UC that compromises the mucosal barrier (Wan et al. 2022). Our study analyzed key markers of the intrinsic apoptotic pathway. The UC group showed a significant shift towards apoptosis, indicated by an increased Bax/Bcl‐2 ratio, increased Cytochrome c release, and elevated levels of initiator (Caspase‐9) and executioner (Caspase‐3) caspases (Figure 7B–E). *L. casei* 39 paraprobiotic treatment significantly counteracted these pro‐apoptotic changes, reducing the Bax/Bcl‐2 ratio, Cytochrome c, Caspase‐9, and Caspase‐3 expression. This robust anti‐apoptotic effect is crucial for preserving epithelial integrity and promoting mucosal healing (Patankar and Becker 2020). Probiotics and paraprobiotics have shown anti‐apoptotic properties in intestinal inflammation (Huang et al. 2023; Teame et al. 2020). These effects are likely intertwined with the impact of paraprobiotics on the PI3K/AKT pathway (which promotes survival), oxidative stress reduction, and anti‐inflammatory effects (Bourebaba et al. 2022; Hyun et al. 2023). Compared to the probiotic, paraprobiotic treatment demonstrated a significantly greater reduction in Caspase‐3 expression (Figure 7D). This result indicates a particularly strong inhibitory effect of the paraprobiotic on the final executioner phase of apoptosis in this model.

Despite the convincing preclinical findings of our study, several limitations should be acknowledged. Our research utilized an acute, chemically induced model of UC; therefore, studies employing chronic models are necessary to evaluate the long‐term efficacy of the treatments and their potential to prevent relapse. Additionally, the results are specific to the *L. casei* 39 strain and the UV‐C inactivation method applied; other strains or inactivation techniques may produce different outcomes. A detailed compositional analysis of the paraprobiotic is warranted to identify its active components. Although key signaling pathways have been identified, further mechanistic studies are required to precisely elucidate the molecular interactions between the paraprobiotic (or its constituents) and host receptors, as well as downstream effectors.

## 5 Conclusion

In conclusion, the *L. casei* 39 paraprobiotic, produced via UV‐C inactivation, demonstrated a comprehensive range of beneficial effects in an acute rat model of UC. It effectively ameliorated clinical signs, modulated local and systemic inflammation, reduced oxidative stress, favorably altered the balance of growth factors VEGF and EGF, suppressed activation of the PI3K/AKT signaling pathway, and mitigated apoptosis. Collectively, these findings highlight the potential of the *L. casei* 39 paraprobiotic as a multi‐target therapeutic agent for UC, impacting key interconnected pathological processes. The observation that the paraprobiotic often exhibited comparable or even superior effects to the live probiotic for specific markers further supports the rationale for developing stable, non‐viable preparations. Future research should focus on validating these findings in chronic UC models, optimizing dosage regimens, and performing comprehensive compositional analyses of the paraprobiotic. Ultimately, translation into human clinical trials is essential to confirm the safety and efficacy of *L. casei* 39 paraprobiotic as a novel therapeutic strategy for UC and to establish its clinical relevance.

## Author Contributions

**Adem Yavaş:** conceptualization, data curation, methodology, supervision, investigation, visualization, formal analysis, and writing original draft. **Ecem Akan:** methodology, formal analysis, and writing – review‐editing.

## Conflicts of Interest

The authors declare no conflicts of interest.

## Data Availability

The data that support the findings of this study are available from the corresponding author upon reasonable request.

## References

[fsn370476-bib-0001] Abdel‐Daim, M. M. , S. M. Farouk , F. F. Madkour , and S. S. Azab . 2015. “Anti‐Inflammatory and Immunomodulatory Effects of Spirulina Platensis in Comparison to *Dunaliella salina* in Acetic Acid‐Induced Rat Experimental Colitis.” Immunopharmacology and Immunotoxicology 37, no. 2: 126–139. 25567297 10.3109/08923973.2014.998368

[fsn370476-bib-0002] Akan, E. , A. Yavaş , and M. Dikme . 2025. “Bioactive Potentials of Paraprobiotic Kefir: Enhanced Protein Hydrolysis and Anticancer Efficacy.” International Dairy Journal 163: 106169.

[fsn370476-bib-0003] Almada, C. N. , C. N. Almada‐Érix , M. S. Bonatto , et al. 2021. “Obtaining Paraprobiotics From Lactobacilus Acidophilus, *Lacticaseibacillus casei* and *Bifidobacterium animalis* Using Six Inactivation Methods: Impacts on the Cultivability, Integrity, Physiology, and Morphology.” Journal of Functional Foods 87: 104826.

[fsn370476-bib-0004] Arda‐Pirincci, P. , and G. Aykol‐Celik . 2020. “Galectin‐1 Reduces the Severity of Dextran Sulfate Sodium (DSS)‐Induced Ulcerative Colitis by Suppressing Inflammatory and Oxidative Stress Response.” Bosnian Journal of Basic Medical Sciences 20, no. 3: 319–328. 31999939 10.17305/bjbms.2019.4539 PMC7416175

[fsn370476-bib-0005] Avci, G. A. , Ü. İ. Yilmaz , and E. Avci . 2024. “Efficacy of Probiotics, Paraprobiotics, and Postbiotics in Colorectal Cancer Cell Line and Their Role in Immune Response.” Revista da Associação Médica Brasileira 70, no. 6: e20240226. 39045970 10.1590/1806-9282.20240226 PMC11288267

[fsn370476-bib-0006] Aydın, B. , T. Çiydem , E. Kaya , and L. Açık . 2021. “Evaluation of the Antioxidant Effects of Postbiotics and Paraprobiotics in Lactic Acid Bacteria Isolated From Traditional Fermented Sausages.” Avrupa Bilim Ve Teknoloji Dergisi 28: 849–852.

[fsn370476-bib-0007] Bourebaba, Y. , K. Marycz , M. Mularczyk , and L. Bourebaba . 2022. “Postbiotics as Potential New Therapeutic Agents for Metabolic Disorders Management.” Biomedicine & Pharmacotherapy 153: 113138. 35717780 10.1016/j.biopha.2022.113138

[fsn370476-bib-0008] Cai, Z. , S. Wang , and J. Li . 2021. “Treatment of Inflammatory Bowel Disease: A Comprehensive Review.” Frontiers in Medicine 8: 765474. 34988090 10.3389/fmed.2021.765474 PMC8720971

[fsn370476-bib-0009] Chen, K. , Y. Li , X. Zhang , R. Ullah , J. Tong , and Y. Shen . 2022. “The Role of the PI3K/AKT Signalling Pathway in the Corneal Epithelium: Recent Updates.” Cell Death & Disease 13, no. 5: 513. 35641491 10.1038/s41419-022-04963-x PMC9156734

[fsn370476-bib-0010] Chen, X. , G. Yang , J.‐H. Song , et al. 2013. “Probiotic Yeast Inhibits VEGFR Signaling and Angiogenesis in Intestinal Inflammation.” PLoS One 8, no. 5: e64227. 23675530 10.1371/journal.pone.0064227 PMC3652827

[fsn370476-bib-0011] Côco, L. Z. , E. de Souza Belisário , E. C. Vasquez , T. M. C. Pereira , R. Aires , and B. P. Campagnaro . 2025. “Probiotics: A Promising Future in the Treatment of Ulcerative Colitis?” Pharmacological Reports 77, no. 3: 1–657. 40214948 10.1007/s43440-025-00724-7

[fsn370476-bib-0012] Cong, Y. , K. Liu , Z. Huang , et al. 2024. “A Bivalent Aptamer‐Based DNA Agonist for EGFR Signaling Effectively Alleviates Ulcerative Colitis In Vivo.” ACS Chemical Biology 19: 1280–1290. 38837175 10.1021/acschembio.4c00098

[fsn370476-bib-0013] Filidou, E. , and G. Kolios . 2021. “Probiotics in Intestinal Mucosal Healing: A New Therapy or an Old Friend?” Pharmaceuticals 14, no. 11: 1181. 34832962 10.3390/ph14111181 PMC8622522

[fsn370476-bib-0014] Gao, J. , B. Cao , R. Zhao , H. Li , Q. Xu , and B. Wei . 2023. “Critical Signaling Transduction Pathways and Intestinal Barrier: Implications for Pathophysiology and Therapeutics.” Pharmaceuticals 16, no. 9: 1216. 37765024 10.3390/ph16091216 PMC10537644

[fsn370476-bib-0015] Gholian, M. M. , A. Babaei , F. Zendeboodi , A. M. Mortazavian , and V. Koushki . 2024. “Effect of Different Inactivation Condition on Lactobacillus Gasseri and *Lactobacillus plantarum* : Culturability, Cell Integrity and Morphology.” LWT 197: 115915.

[fsn370476-bib-0016] He, T. , K. Wang , P. Zhao , et al. 2022. “Integrative Computational Approach Identifies Immune‐Relevant Biomarkers in Ulcerative Colitis.” FEBS Open Bio 12, no. 2: 500–515. 10.1002/2211-5463.13357 PMC8804607 34939750

[fsn370476-bib-0017] Huang, C. , W. Hao , X. Wang , R. Zhou , and Q. Lin . 2023. “Probiotics for the Treatment of Ulcerative Colitis: A Review of Experimental Research From 2018 to 2022.” Frontiers in Microbiology 14: 1211271. 37485519 10.3389/fmicb.2023.1211271 PMC10358780

[fsn370476-bib-0018] Hyun, J.‐H. , I.‐K. Woo , K.‐T. Kim , et al. 2023. “Heat‐Treated Paraprobiotic Latilactobacillus Sakei KU15041 and Latilactobacillus Curvatus KU15003 Show an Antioxidant and Immunostimulatory Effect.” Journal of Microbiology and Biotechnology 34, no. 2: 358–366. 37997261 10.4014/jmb.2309.09007 PMC10940752

[fsn370476-bib-0019] Jadhav, A. , S. Jagtap , S. Vyavahare , A. Sharbidre , and B. Kunchiraman . 2023. “Reviewing the Potential of Probiotics, Prebiotics and Synbiotics: Advancements in Treatment of Ulcerative Colitis.” Frontiers in Cellular and Infection Microbiology 13: 1268041. 38145046 10.3389/fcimb.2023.1268041 PMC10739422

[fsn370476-bib-0020] Jalil, A. T. , N. F. Hassan , S. J. Abdulameer , et al. 2023. “Phosphatidylinositol 3‐Kinase Signaling Pathway and Inflammatory Bowel Disease: Current Status and Future Prospects.” Fundamental and Clinical Pharmacology 37, no. 5: 910–917. 36939850 10.1111/fcp.12894

[fsn370476-bib-0021] Kanazawa, S. , T. Tsunoda , E. Onuma , T. Majima , M. Kagiyama , and K. Kikuchi . 2001. “VEGF, Basic‐FGF, and TGF‐β in Crohn's Disease and Ulcerative Colitis: A Novel Mechanism of Chronic Intestinal Inflammation.” American Journal of Gastroenterology 96, no. 3: 822–828. 11280558 10.1111/j.1572-0241.2001.03527.x

[fsn370476-bib-0022] Kang, Y. Y. , H. J. Song , S. Y. Park , et al. 2025. “Comparative Effects of Probiotics and Paraprobiotics Derived From Lactiplantibacillus Plantarum, Latilactobacillus Sakei, and Limosilactobacillus Reuteri in a DSS‐Induced Ulcerative Colitis Mouse Model.” Journal of Microbiology and Biotechnology 35: e2411045. 40016142 10.4014/jmb.2411.11045 PMC11896797

[fsn370476-bib-0023] Kye, Y. J. , S. Y. Lee , H. R. Kim , et al. 2022. “ *Lactobacillus acidophilus* PIN7 Paraprobiotic Supplementation Ameliorates DSS‐Induced Colitis Through Anti‐Inflammatory and Immune Regulatory Effects.” Journal of Applied Microbiology 132, no. 4: 3189–3200. 34878713 10.1111/jam.15406

[fsn370476-bib-0024] Lai, W. , C. Xian , M. Chen , et al. 2023. “Single‐Cell and Bulk Transcriptomics Reveals M2d Macrophages as a Potential Therapeutic Strategy for Mucosal Healing in Ulcerative Colitis.” International Immunopharmacology 121: 110509. 37369160 10.1016/j.intimp.2023.110509

[fsn370476-bib-0025] Lam, E. K. , L. Yu , H. P. Wong , et al. 2007. “Probiotic *Lactobacillus rhamnosus* GG Enhances Gastric Ulcer Healing in Rats.” European Journal of Pharmacology 565, no. 1–3: 171–179. 17395175 10.1016/j.ejphar.2007.02.050

[fsn370476-bib-0026] Lê, A. , M. Mantel , J. Marchix , M. Bodinier , G. Jan , and M. Rolli‐Derkinderen . 2022. “Inflammatory Bowel Disease Therapeutic Strategies by Modulation of the Microbiota: How and When to Introduce Pre‐, Pro‐, Syn‐, or Postbiotics?” American Journal of Physiology. Gastrointestinal and Liver Physiology 323, no. 6: G523–G553. 36165557 10.1152/ajpgi.00002.2022

[fsn370476-bib-0027] Lee, H.‐J. , M. T. H. Tran , M. H. Le , E. E. Justine , and Y.‐J. Kim . 2024. “Paraprobiotic Derived From *Bacillus velezensis* GV1 Improves Immune Response and Gut Microbiota Composition in Cyclophosphamide‐Treated Immunosuppressed Mice.” Frontiers in Immunology 15: 1285063. 38455053 10.3389/fimmu.2024.1285063 PMC10918466

[fsn370476-bib-0028] Li, K. , M. Yang , M. Tian , et al. 2024. “The Preventive Effects of *Lactobacillus casei* 03 on *Escherichia coli*‐Induced Mastitis In Vitro and In Vivo.” Journal of Inflammation 21, no. 1: 5. 38395896 10.1186/s12950-024-00378-x PMC10893599

[fsn370476-bib-0029] Liang, D. , F. Wu , D. Zhou , B. Tan , and T. Chen . 2024. “Commercial Probiotic Products in Public Health: Current Status and Potential Limitations.” Critical Reviews in Food Science and Nutrition 64, no. 19: 6455–6476. 36688290 10.1080/10408398.2023.2169858

[fsn370476-bib-0030] Liashev, A. Y. , G. S. Mal , A. V. Solin , and M. A. Balanina . 2024. “The Effect of Dalargin on Growth Factors Content in Experimental Ulcerative Colitis.” Research Results in Pharmacology 10, no. 1: 67–73.

[fsn370476-bib-0031] Liu, C. , X. Qi , D. Li , et al. 2024. “Limosilactobacillus Fermentum HF06‐Derived Paraprobiotic and Postbiotic Alleviate Intestinal Barrier Damage and Gut Microbiota Disruption in Mice With Ulcerative Colitis.” Journal of the Science of Food and Agriculture 104, no. 3: 1702–1712. 37851615 10.1002/jsfa.13057

[fsn370476-bib-0032] Manna, A. , A. Chakraborty , S. Bag , S. Chackrabarty , and B. R. Basu . 2024. “Postbiotics: A New Post in Biotics and Its Prospective Role in Human Health and Diseases.” International Journal of Pharmaceutical Research and Applications 9, no. 3: 2067–2082.

[fsn370476-bib-0033] Martyniak, A. , A. Medyńska‐Przęczek , A. Wędrychowicz , S. Skoczeń , and P. J. Tomasik . 2021. “Prebiotics, Probiotics, Synbiotics, Paraprobiotics and Postbiotic Compounds in IBD.” Biomolecules 11, no. 12: 1903. 34944546 10.3390/biom11121903 PMC8699341

[fsn370476-bib-0034] Ning, H. , J. Liu , J. Tan , M. Yi , and X. Lin . 2024. “The Role of the Notch Signalling Pathway in the Pathogenesis of Ulcerative Colitis: From the Perspective of Intestinal Mucosal Barrier.” Frontiers in Medicine 10: 1333531. 38249980 10.3389/fmed.2023.1333531 PMC10796567

[fsn370476-bib-0035] Ozal‐Coskun, C. , E. F. Karaman , S. Ozden , E. Kaptan , and P. Arda . 2024. “The Role of Sodium Phenylbutyrate and Suramin in the Prevention of Ulcerative Colitis Induced by Dextran Sulfate Sodium.”

[fsn370476-bib-0036] Patankar, J. V. , and C. Becker . 2020. “Cell Death in the Gut Epithelium and Implications for Chronic Inflammation.” Nature Reviews Gastroenterology & Hepatology 17, no. 9: 543–556. 32651553 10.1038/s41575-020-0326-4

[fsn370476-bib-0037] Pompili, S. , G. Latella , E. Gaudio , R. Sferra , and A. Vetuschi . 2021. “The Charming World of the Extracellular Matrix: A Dynamic and Protective Network of the Intestinal Wall.” Frontiers in Medicine 8: 610189. 33937276 10.3389/fmed.2021.610189 PMC8085262

[fsn370476-bib-0038] Quansah, E. , E. Gardey , A. Ramoji , et al. 2023. “Intestinal Epithelial Barrier Integrity Investigated by Label‐Free Techniques in Ulcerative Colitis Patients.” Scientific Reports 13, no. 1: 2681. 36792686 10.1038/s41598-023-29649-y PMC9931702

[fsn370476-bib-0039] Ramani, A. , R. Deshmukh , R. Seth , K. Gandhi , R. Sharma , and V. Sharma . 2023. “Paraprobiotics in the Dairy Industry: Current Research and Future Prospects: A Review.” Bhartiya Krishi Anusandhan Patrika 38, no. 2: 124–129.

[fsn370476-bib-0040] Saedi, S. , S. Derakhshan , A. Hasani , et al. 2025. “Recent Advances in Gut Microbiome Modulation: Effect of Probiotics, Prebiotics, Synbiotics, and Postbiotics in Inflammatory Bowel Disease Prevention and Treatment.” Current Microbiology 82, no. 1: 1–13. 10.1007/s00284-024-03997-y 39589525

[fsn370476-bib-0041] Segal, J. P. , J.‐F. LeBlanc , and A. L. Hart . 2021. “Ulcerative Colitis: An Update.” Clinical Medicine 21, no. 2: 135–139. 33762374 10.7861/clinmed.2021-0080 PMC8002778

[fsn370476-bib-0042] Shahid, M. , M. Raish , A. Ahmad , et al. 2022. “Sinapic Acid Ameliorates Acetic Acid‐Induced Ulcerative Colitis in Rats by Suppressing Inflammation, Oxidative Stress, and Apoptosis.” Molecules 27, no. 13: 4139. 35807383 10.3390/molecules27134139 PMC9268465

[fsn370476-bib-0043] Song, S. , A. Jeong , J. Lim , B. K. Kim , D. J. Park , and S. Oh . 2023. “Lactiplantibacillus Plantarum L67 Probiotics vs Paraprobiotics for Reducing Pro‐Inflammatory Responses in Colitis Mice.” International Journal of Dairy Technology 76, no. 1: 168–177.

[fsn370476-bib-0044] Stefani, C. , D. Miricescu , I.‐I. Stanescu‐Spinu , et al. 2021. “Growth Factors, PI3K/AKT/mTOR and MAPK Signaling Pathways in Colorectal Cancer Pathogenesis: Where Are We Now?” International Journal of Molecular Sciences 22, no. 19: 10260. 34638601 10.3390/ijms221910260 PMC8508474

[fsn370476-bib-0045] Štofilová, J. , M. Kvaková , A. Kamlárová , E. Hijová , I. Bertková , and Z. Guľašová . 2022. “Probiotic‐Based Intervention in the Treatment of Ulcerative Colitis: Conventional and New Approaches.” Biomedicine 10, no. 9: 2236. 10.3390/biomedicines10092236 PMC9496552 36140337

[fsn370476-bib-0046] Tan, L. , J. Fu , F. Feng , et al. 2020. “Engineered Probiotics Biofilm Enhances Osseointegration via Immunoregulation and Anti‐Infection.” Science Advances 6, no. 46: eaba5723. 33188012 10.1126/sciadv.aba5723 PMC10763977

[fsn370476-bib-0047] Tarnawski, A. S. , and A. Ahluwalia . 2021. “The Critical Role of Growth Factors in Gastric Ulcer Healing: The Cellular and Molecular Mechanisms and Potential Clinical Implications.” Cells 10, no. 8: 1964. 34440733 10.3390/cells10081964 PMC8392882

[fsn370476-bib-0048] Teame, T. , A. Wang , M. Xie , et al. 2020. “Paraprobiotics and Postbiotics of Probiotic Lactobacilli, Their Positive Effects on the Host and Action Mechanisms: A Review.” Frontiers in Nutrition 7: 570344. 33195367 10.3389/fnut.2020.570344 PMC7642493

[fsn370476-bib-0049] Terzaghi, B. E. , and W. Sandine . 1975. “Improved Medium for Lactic Streptococci and Their Bacteriophages.” Applied Microbiology 29, no. 6: 807–813. 16350018 10.1128/am.29.6.807-813.1975 PMC187084

[fsn370476-bib-0050] Towbin, H. , T. Staehelin , and J. Gordon . 1979. “Electrophoretic Transfer of Proteins From Polyacrylamide Gels to Nitrocellulose Sheets: Procedure and Some Applications.” Proceedings of the National Academy of Sciences 76, no. 9: 4350–4354. 10.1073/pnas.76.9.4350 PMC411572 388439

[fsn370476-bib-0051] Vaidyanathan, L. 2021. “Growth Factors in Wound Healing—A Review.” Biomed Pharmacol J 14, no. 3: 1469–1480.

[fsn370476-bib-0052] Villablanca, E. J. , K. Selin , and C. R. Hedin . 2022. “Mechanisms of Mucosal Healing: Treating Inflammatory Bowel Disease Without Immunosuppression?” Nature Reviews Gastroenterology & Hepatology 19, no. 8: 493–507. 35440774 10.1038/s41575-022-00604-y

[fsn370476-bib-0053] Wan, Y. , L. Yang , S. Jiang , D. Qian , and J. Duan . 2022. “Excessive Apoptosis in Ulcerative Colitis: Crosstalk Between Apoptosis, ROS, ER Stress, and Intestinal Homeostasis.” Inflammatory Bowel Diseases 28, no. 4: 639–648. 34871402 10.1093/ibd/izab277

[fsn370476-bib-0054] Wang, L. , J. Xu , P. Xue , et al. 2021. “Thermo‐Sensitive Hydrogel With Mussel‐Inspired Adhesion Enhanced the Non‐Fibrotic Repair Effect of EGF on Colonic Mucosa Barrier of TNBS‐Induced Ulcerative Colitis Rats Through Macrophage Polarizing.” Chemical Engineering Journal 416: 129221.

[fsn370476-bib-0055] Wu, D. , M. Cao , J. Zhou , S. Yan , and J. Peng . 2021. “ *Lactobacillus casei* T1 From Kurut Against *Helicobacter pylori*‐Induced Inflammation and the Gut Microbial Disorder.” Journal of Functional Foods 85: 104611.

[fsn370476-bib-0056] Xu, Z. , X. Zhang , R. Lu , et al. 2022. “Mechanism of Fructus Mume Pills Underlying Their Protective Effects in Rats With Acetic Acid‐Inducedulcerative Colitis via the Regulation of Inflammatory Cytokines and the VEGF‐PI3K/Akt‐eNOS Signaling Pathway.” Evidence‐Based Complementary and Alternative Medicine 2022: 4621131. 35620404 10.1155/2022/4621131 PMC9129976

[fsn370476-bib-0057] Yan, L. , C. Gu , S. Gao , and B. Wei . 2023. “Epigenetic Regulation and Therapeutic Strategies in Ulcerative Colitis.” Frontiers in Genetics 14: 1302886. 38169708 10.3389/fgene.2023.1302886 PMC10758477

[fsn370476-bib-0058] Zawistowska‐Rojek, A. , and S. Tyski . 2022. “How to Improve Health With Biological Agents—Narrative Review.” Nutrients 14, no. 9: 1700. 35565671 10.3390/nu14091700 PMC9103441

[fsn370476-bib-0059] Zhang, H. , F. Zhang , and W. Li . 2021. “Function of Intestinal Barrier Protected by Regulating the miR‐199a‐3p in Ulcerative Colitis: Modulation of IL‐23/IL‐17A Axis.” Fundamental & Clinical Pharmacology 35, no. 5: 852–860. 33475196 10.1111/fcp.12650

[fsn370476-bib-0060] Zhang, W. , Y.‐H. Zhu , J.‐C. Yang , G.‐Y. Yang , D. Zhou , and J.‐F. Wang . 2015. “A Selected *Lactobacillus rhamnosus* Strain Promotes EGFR‐Independent Akt Activation in an Enterotoxigenic *Escherichia coli* K88‐Infected IPEC‐J2 Cell Model.” PLoS One 10, no. 4: e0125717. 25915861 10.1371/journal.pone.0125717 PMC4411159

[fsn370476-bib-0061] Zhang, X. , F. Zhang , Y. Li , et al. 2024. “Blockade of PI3K/AKT Signaling Pathway by Astragaloside IV Attenuates Ulcerative Colitis via Improving the Intestinal Epithelial Barrier.” Journal of Translational Medicine 22, no. 1: 406. 38689349 10.1186/s12967-024-05168-w PMC11061986

[fsn370476-bib-0062] Zhu, F. , J. Zheng , F. Xu , Y. Xi , J. Chen , and X. Xu . 2021. “Resveratrol Alleviates Dextran Sulfate Sodium‐Induced Acute Ulcerative Colitis in Mice by Mediating PI3K/Akt/VEGFA Pathway.” Frontiers in Pharmacology 12: 693982. 34497510 10.3389/fphar.2021.693982 PMC8419259

[fsn370476-bib-0063] Zoroddu, S. , B. Di Lorenzo , P. Paliogiannis , A. A. Mangoni , C. Carru , and A. Zinellu . 2025. “Vascular Endothelial Growth Factor in Inflammatory Bowel Disease: A Systematic Review and Meta‐Analysis.” European Journal of Clinical Investigation 55, no. 3: e14361. 39545600 10.1111/eci.14361 PMC11810564

